# A novel design and analysis of hybrid fuzzy logic MPPT controller for solar PV system under partial shading conditions

**DOI:** 10.1038/s41598-024-60870-5

**Published:** 2024-05-04

**Authors:** Sunkara Sunil Kumar, K. Balakrishna

**Affiliations:** Vignan’s Foundation for Science Technology and Research, Vadlamudi, 522213 India

**Keywords:** Boost power converter, Duty value, Fast system response, High voltage gain, MPP tracking speed, System efficiency, Plus less oscillations of MPP, Engineering, Electrical and electronic engineering

## Abstract

Renewable energy resources are more useful when associated with the thermal power generation network because of their high accessibility in the environment, good system response, easy manufacturing, plus high scalable. So, the present research is going on solar power to reduce consumer grid dependency. The running of the PV network is quite easier, plus less human sources are involved. However, the solar modules’ power generation is nonlinear fashion. So, the collection of peak power from the sunlight-dependent systems is a highly challenging task. In this article, a Modified Differential Step Grey Wolf Optimization with Adaptive Fuzzy Logic Controller (MDSGWO with FLC) is developed for collecting the maximum power from renewable energy resources under diverse Partial Shading Conditions (PSCs). The introduced method comprehensive analysis has been done along with the other recently existing MPPT methods in terms of convergence speed, MPP tracking accuracy, operating efficiency of the introduced method, functioning duty value of the DC–DC boost power converter, dependence of MPPT on sunlight system, total number of sensing devices are needed, plus peak power extraction from the proposed system. Here, the sunlight power generation cost is more to limit this issue, a power converter is selected in the second objective to develop the voltage source capability of the PV network. The overall PV-interfaced power converter network is examined by utilizing the MATLAB environment.

## Introduction

From the present nonrenewable power generation strategy, the utilization of thermal, nuclear, plus natural gas systems is reducing drastically. In addition, the gas-dependent power supply networks also work as a nonrenewable energy source because its drawbacks are more effect on the environmental conditions, high greenhouse gas emissions, more development cost, high complexity in input fuel transportation, less flexibility, plus less robustness. So, the present distribution networks are working on renewable energy systems to enhance the power supply flexibility to the local consumers. The classifications of renewable power supply systems are geothermal, hydropower systems, wind power systems, tidal networks, biofuels, plus sunlight power supply systems. In^1^, the researchers discussed the geothermal systems which transform water energy into steam energy for running the steam turbines thereby the generator starts running at different speeds to generate the electricity. The features of geothermal power networks require high operating temperatures, more groundwater requirements, plus less reliability. However, the geothermal systems create greenhouse gas emissions under the earth thereby temperature rises on the earth^2^. So, the ocean power supply networks are applied to the microgrid network for supplying the power to the rural area human beings. Here, the tidal energy is transferred to electrical energy by using the bidirectional generators. These tidal systems can supply a high amount of power to industries when associated with geothermal power networks.

The wind power supply networks are utilized in^3^ to limit the disadvantages of geothermal, plus tidal power supply systems. Here, the wind turbines capture the wind velocity and the bidirectional generator utilizes the wind velocity for supplying the power to the emergency conditions. The wind systems create more economic growth and provide more jobs to human beings. The features of this system are cost-effective, plus work in many directions. But the wind systems supply discontinuity power and construction of this system creates local disturbances, is highly dangerous to wildlife, plus gives little noise. So, hydropower networks are used in place of wind systems to limit the drawbacks of wind power supply networks. In these hydro systems, the head of water has been playing a major role in peak load demand conditions^4^. To increase the water head level, there are various types of water storage dams constructed near hilly areas to supply constant power to urban human beings. The merits of hydropower networks are irrigation support, clean drinking water, plus flood control. In addition, hydro systems provide low-cost electricity and durability over time when associated with other renewable power sources. However, the failure of dams creates widespread flooding, destruction of property, and the possibility of more civilian deaths^5^.

**Figure 1:**
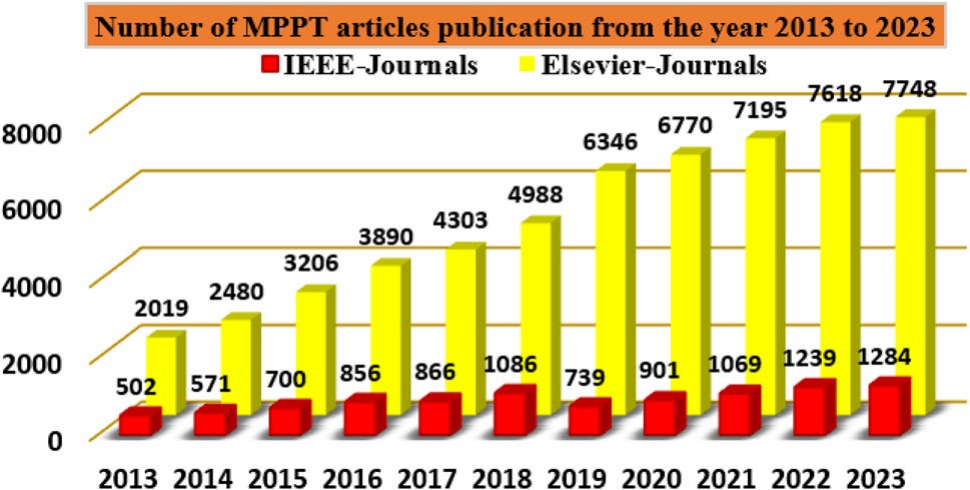
Publication analysis of various MPPT methods.

**Figure 2:**
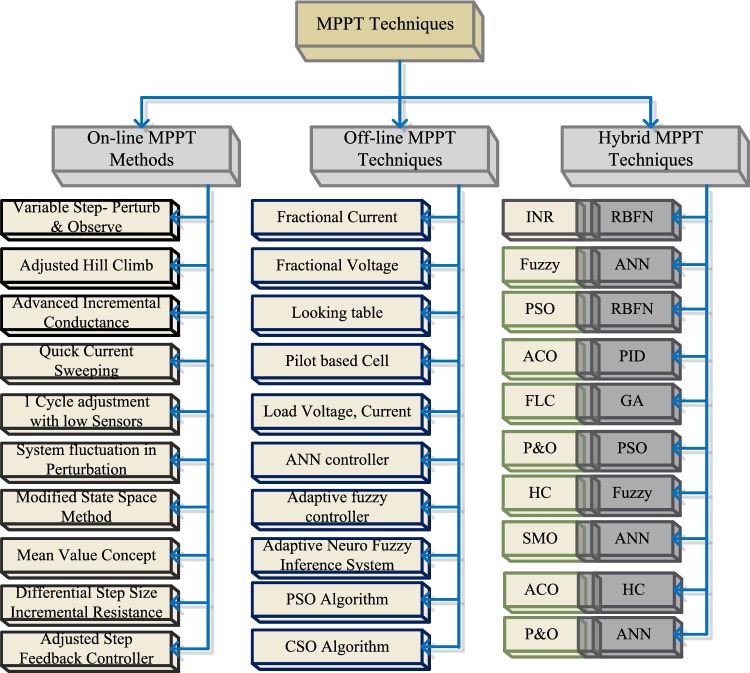
Detailed differentiation of different power point tracking controllers.

So, the present power generation industries are moving towards the sunlight systems because easy to handle, have less impact on human health, have zero emissions, provide more economic growth, plus more efficient for rural areas peoples. A single sunlight PV cell provides 0.83 to 0.94 V which may not be useful for any consumer applications. So, the sunlight system manufactures and integrates the PV cells in a series fashion to enhance the potential supply capability of the sunlight system. Similarly, the PV cells are integrated in a parallel fashion to meet the high-rating current demand. From the recently existing articles, the power flow in the PV cell is same to the normal P–N diodes. The PV manufacturing technologies are polycrystalline, monocrystalline, plus thin film. Among all the manufacturing techniques, thin film provides more efficiency. However, the per unit generation cost of sunlight system is very high to limit this issue, there are different types of PV cell development methodologies applied to the sunlight networks which are 1-diode, two-diode, plus 3-diodes solar cells. In^6^, the authors studied the PV cell technologies in terms of fill factor, plus peak voltage extraction. From the simulative results analysis, the authors concluded that the 3-diode PV sunlight system provides more accurate nonlinear characteristics and more functioning efficiency. So, in this article, a triple-diode PV circuit is used to develop the PV module, and it supplies nonlinear power to the load. As a result, the extraction of more power from the sunlight network is quite a difficult task. The Maximum Power Point Tracking (MPPT) concept is selected in the article^7^ for running the functioning point of the sunlight system near the actual working point of the PV, and its publication record from the last few years is illustrated in Fig. 1. The detailed differentiation of PV-based MPPT controllers is mentioned in Fig. 2.

From the literature study, the available power point tracking methods are artificial intelligence, data science, soft computing, nature-inspired algorithms, plus conventional controllers. In^8^, the authors discussed the Modified Perturb and Observe (MP&O) concept for selecting the proper duty cycle for the quadratic converter. This quadratic converter circuit is connected to the DC link of the smart grid network to enhance the voltage supply capability of the smart grid network. In this method, the power is utilized as a reference parameter for extracting the peak power from the sunlight system. The merits of this popular P&O controller are very easy to develop, has low implementation cost, has quick convergence, plus more reliable. However, this method may not be useful for highly accurate sunlight systems. Also, this controller produces more oscillations across the functioning point of the sunlight network. The disadvantages of P&O are limited by selecting the Incremental Conductance Method (ICM) to reduce the power loss in the system by equating the load resistance with the source impedance. Here, the conductance of the PV is adjusted until the introduced system works at high efficiency. The demerits of ICM are more design cost when associated with the basic P&O controller, plus more complexity in the controller circuit. The sliding model is designed in^9^ for sunlight power supply networks to make the overall system work under any atmospheric conditions. This controller is more suitable for partially shaded sunlight systems. The major problem of this controller is the high execution cost.

Most of the sunlight systems consist of power electronics devices and their input irradiations are also not constant. Due to these reasons, the sunlight system suffers from distortions in the PV power. The distortions of PV voltage, plus solar currents are sent to the Kalman filter for suppressing the harmonics in the PV system thereby generating the switching pulses to the power converter^10^. This filter controller reduces the overall system size, plus optimizes the heat conduction losses of the converter. This Kalman controller is useful only for constant sunlight temperature, and irradiation values. This controller takes high convergence time, plus requires more mathematical calculations. The fractional current, plus fractional power methodologies are completely approximated methods that are applied for street light applications. These methods needed additional power semiconductor devices for identifying the short-circuit current, and PV open circuit voltage. Also, these controllers develop the overall sunlight system complexity. So, the researchers working on the convolution neural networks for optimizing the tracking time of the sunlight system MPP. The features of convolution neural systems are automatic feature extraction, no need for human supervision, more accuracy for local MPP identification, the possibility of weight sharing, optimal computation needed, the ability to solve big data sets, plus hierarchical learning. However, convolution neural networks are less applicable to the fast changes of atmospheric conditions, and they need a large memory footprint, plus less effectiveness for the sequential data. Also, it takes training time for a huge amount of data^11^.

So, the deep learning neural networks (DLNN) are introduced in the article^12^ to recognize the partial shading conditions of the sunlight systems. The DLNNs have the possibility of solving any nonlinear issue without any complexity. The DLNNs collect the sunlight temperature, ideality factors data, sunlight irradiations, plus available solar power for sending the switching pulses to the quadratic converter. The merits of DLNN are efficient processing of unstructured data, hidden relationships, easy pattern discovery, unsupervised learning, plus Volatile data processing. The disadvantages of deep learning neural networks are more computational cost, lack of interpretability, plus overfitting. So, the researchers move towards the radial basis functional network algorithms (RBFNA) for tracking the global point of the sunlight system under various shading pattern values. The RBFNA is a real-valued function and its value is completely depending on the distance between the input point, plus the fixed point. This algorithm is best suitable for function approximation issues^13^. The features of RBFNA are good generalization, more flexibility in design, the best online learning capability, plus high tolerance to input noise. However, these networks needed good coverage of input space.

**Figure 3:**
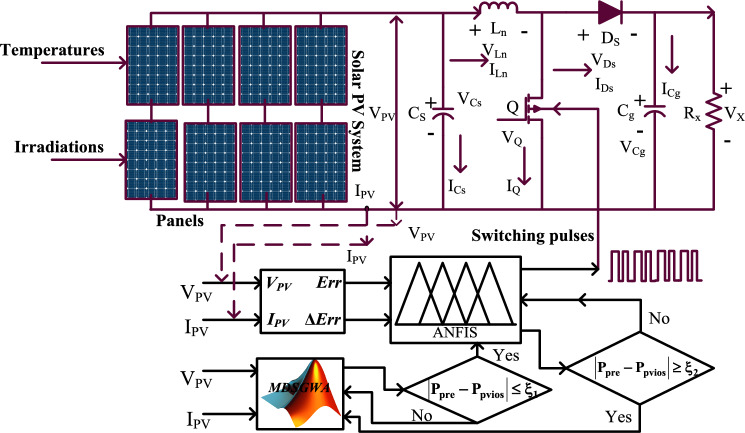
Proposed modified grey wolf technique for ANFIS power point tracking identifier.

So, in this article, a hybrid Modified Different Step Grey Wolf Technology (MDSGWT) is selected for the identification of membership functions of the Fuzzy Logic Controller. The merits of this hybrid MPPT controller are few sensing devices are needed, a low value of iterations is needed for identifying the optimal solution, fast tracking speed of the functioning point of the sunlight system, plus less settling time of the sunlight output power. The sunlight system’s foremost issue is a low level of voltage supply which is increased from a low-level voltage to a high-level voltage to meet the rural people's load demands. There are different DC–DC converter circuit types available in the literature study which are flyback, plus feedforward isolated power converters. These types of DC–DC converters need additional power electronics circuits to improve the sunlight system voltage, and the utilization of a transformer in the converter circuit improves the system size thereby circuit complexity, and its implementation cost are increased when associated with the non-isolated power electronic circuits^14^. Due to these demerits, the single-switch power converter circuit is used in this work to enhance the voltage rating of the sunlight system. The facts of this converter circuit are low design, plus implementation cost, less component utilization, simple structure, plus more flexibility. The working converter circuit and its switching pulse generation are illustrated in Fig. 3.

## Mathematical analysis 3-diode circuit PV cell

The manufacturers use different types of PV technologies for developing the sunlight system. From the previously available data, the sunlight systems may be designed either by applying a 1-diode PV circuit or a 2-diode PV circuit. The 1-diode PV circuit development has been done by using the five major constraints which are demonstrated as short-circuited PV array current (I_SPC_), open-circuited PV system voltage (V_OCPV_), maximum sunlight network power (P_PP_), peak available voltage of the sunlight PV module (P_PV_), plus utilized P–N diode ideality factor (δ). This one diode-dependent PV circuit is a completely approximated sunlight cell. In this PV sunlight system, the reverse recombination of the charging effect is neglected. So, this one diode circuit model sunlight system may not generate more accurate V–I, plus P–V nonlinear characteristics. Also, it reduces the functioning efficiency of the overall sunlight network. To limit these many issues of the one-diode sunlight system, the 2-diode sunlight technology is developed in the article^15^ for collecting the recombination effect of the semiconductor diode. Due to the additional component in the sunlight system, the development cost of the network and its circuit design complexity are increased. Also, there are a few additional parameters extraction is needed in this 2-diode sunlight system when associated with the one-diode model.

**Figure 4:**
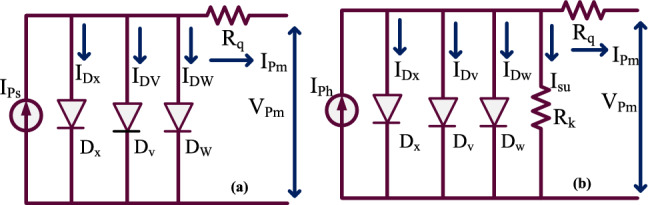
3-diode related sunlight power network, (**a**) absence of shunt resistive element, plus (**b**) presence of shunt resistive element.

The 2-diode sunlight system supplies flexible and accurate electricity to the water storage systems. The variables required for the development of this double-diode solar circuit are seven which are identified as P_PV_, I_SPCt_, I_SPCy_, V_OCPV_, P_PP_, ${\updelta }_{{\text{x}}},$ plus ${\updelta }_{{\text{C}}}$. However, this two-diode PV network suffers from leakage currents. In this work, the triple diode sunlight system is proposed for covering the junction recombination effects, plus leakage currents of the PV modules. The detailed 3-diode circuit by utilizing the nine parameters is discussed in Fig. 4a, plus Fig. 4b. From Fig. 4a, the shunt resistance in the circuit is absent the sunlight system generated current is mentioned in Eq. (1), and the shunt resistive element is not considered because of the assumption that the manufactured PV is very perfect. The shunt resistance is considered in Fig. 4b for removing the reverse leakage currents in the system. Here, the modified wind-driven optimization methodology is selected for the nine parameters of the proposed system which are illustrated as I_SPCt_, I_SPCy_, I_SPCu_, V_OCPV_, δ_x_, δ_v_, δ_w_, R_k_, plus R_q_.

1 $${{\text{I}}}_{{\text{Pm}}}={{\text{I}}}_{{\text{Ps}}}-{{\text{I}}}_{{{\text{D}}}_{{\text{x}}}}-{{\text{I}}}_{{{\text{D}}}_{{\text{v}}}}-{{\text{I}}}_{{{\text{D}}}_{{\text{w}}}}$$

2 $${{\text{I}}}_{{\text{Pm}}}={{\text{I}}}_{{\text{Ps}}}-{{\text{I}}}_{{\text{SPVC}}\_{\text{x}}}\left({{\text{e}}}^{\frac{{\text{q}}*({{\text{V}}}_{{\text{Pm}}}+{{\text{I}}}_{{\text{Pm}}}*{{\text{R}}}_{{\text{q}}})}{{\updelta }_{{\text{x}}}*{\text{K}}*{\text{T}}}}-1\right)-{{\text{I}}}_{{\text{SPVC}}\_{\text{v}}}\left({{\text{e}}}^{\frac{{\text{q}}({{\text{V}}}_{{\text{Pm}}}+{{\text{I}}}_{{\text{Pm}}}*{{\text{R}}}_{{\text{q}}})}{{\updelta }_{{\text{v}}}*{\text{K}}*{\text{T}}}}-1\right)-{\mathrm{ I}}_{{\text{SPVC}}\_{\text{w}}}\left({{\text{e}}}^{\frac{{\text{q}}({{\text{V}}}_{{\text{Pm}}}+{{\text{I}}}_{{\text{Pm}}}*{{\text{R}}}_{{\text{q}}})}{{\updelta }_{{\text{w}}}*{\text{K}}*{\text{T}}}}-1\right)$$

3 $${{\text{I}}}_{{\text{Ps}}}={{\text{I}}}_{{\text{Pm}}}-{{\text{I}}}_{{\text{Dx}}}-{{\text{I}}}_{{\text{Dv}}}-{{\text{I}}}_{{\text{Dw}}}-{{\text{I}}}_{{\text{k}}}$$

4 $${{\text{I}}}_{{\text{Pm}}}={{\text{I}}}_{{\text{Ps}}}-{{\text{I}}}_{{\text{SPVC}}\_{\text{x}}}\left({{\text{e}}}^{\frac{{\text{q}}*({{\text{V}}}_{{\text{Pm}}}+{{\text{I}}}_{{\text{Pm}}}*{{\text{R}}}_{{\text{q}}})}{{\updelta }_{{\text{x}}}*{\text{K}}*{\text{T}}}}-1\right)-{{\text{I}}}_{{\text{SPVC}}\_{\text{v}}}\left({{\text{e}}}^{\frac{{\text{q}}({{\text{V}}}_{{\text{Pm}}}+{{\text{I}}}_{{\text{Pm}}}{{\text{R}}}_{{\text{q}}})}{{\updelta }_{{\text{v}}}*{\text{K}}*{\text{T}}}}-1\right)-{{\text{I}}}_{{\text{b}}}$$

5 $${{\text{I}}}_{{\text{Ps}}}={{\text{I}}}_{{\text{SPVC}}\_{\text{w}}}\left({{\text{e}}}^{\frac{{\text{q}}({{\text{V}}}_{{\text{Pm}}}+{{\text{I}}}_{{\text{Pm}}}*{{\text{R}}}_{{\text{q}}})}{{\updelta }_{{\text{w}}}{\text{K}}*{\text{T}}}}-1\right)+\frac{{{\text{V}}}_{{\text{Pm}}}+{{\text{I}}}_{{\text{Pm}}}{{\text{R}}}_{{\text{q}}}}{{{\text{R}}}_{{\text{k}}}}$$

6 $${{\text{I}}}_{{\text{SPVC}}\_{\text{x}}}={{\text{I}}}_{{\text{SPVC}}\_{\text{v}}}={{\text{I}}}_{{\text{SPVC}}\_{\text{w}}}={{\text{I}}}_{{\text{on}}}* (\frac{{\text{T}}}{{{\text{T}}}_{{\text{n}}}}{)}^{3}{\mathrm{ e}}^{\frac{{\text{q}}*{\text{Eg}}}{{\text{nk}}}\left(\frac{1}{{{\text{T}}}_{{\text{n}}}}-\frac{1}{{\text{T}}}\right)}$$

7 $${{\text{I}}}_{{\text{on}}}={{\text{I}}}_{{\text{on}}\_{\text{x}}}={{\text{I}}}_{{\text{on}}\_{\text{v}}}={{\text{I}}}_{{\text{on}}\_{\text{w}}}=\frac{{{\text{I}}}_{{\text{SPVC}}\_{\text{n}}}}{{{\text{e}}}^{\left(\frac{{{\text{V}}}_{{\text{oc}}\_{\text{n}}}}{\updelta *{{\text{V}}}_{*{\text{Tn}}}}\right)}}$$

### Analysis of partial-shaded solar PV modules

**Figure 5:**
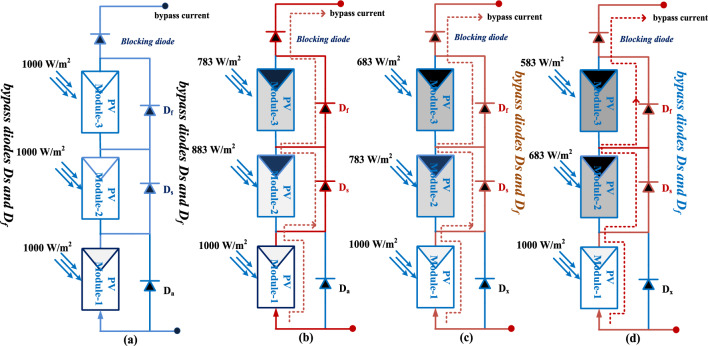
Sunlight capturing of different solar modules with various irradiation values.

**Figure 6:**
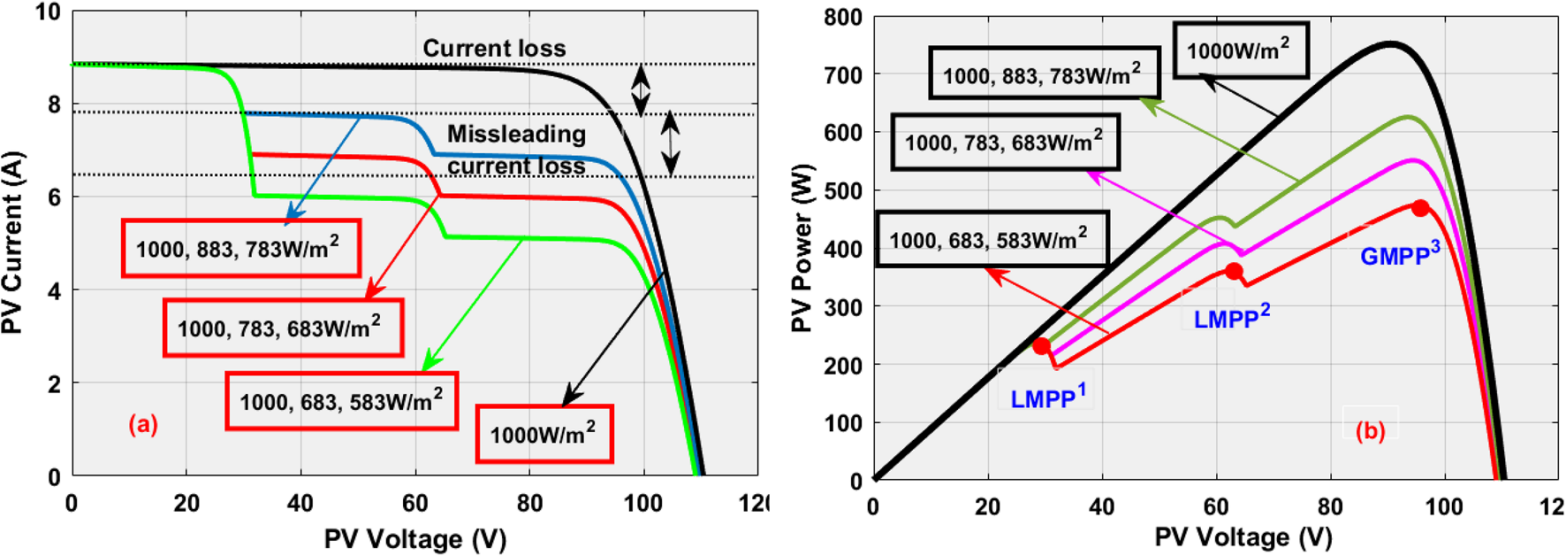
Available sunlight-based solar system I–V & P–V characteristics.

Generally, the sunlight modules are placed near to shadow-free place. However, the tall building shadow, clouds plus trees affect the sunlight system performance by nonuniform irradiation conditions. Due to this nonuniform sunlight irradiation, the shaded sunlight modules observe the electricity from the nonshaded sunlight PV modules which is discussed in Fig. 5. From Fig. 5, the sunlight incident on the PV string varies from time to time because the sunlight source is maximum at midday, and it is very low at morning and evening. Also, the incident angle of sunlight in the morning is less. So, the sunlight collection of the PV string is low. In the afternoon, the sunlight incident angle is high thereby power generation of the PV network is more. Here, the power semiconductor device is placed across each PV cell to limit the overflow currents of the sunlight network. From Fig. 5a, the sunlight incident on the PV string is constant concerning time, and it is rotated continuously to collect maximum sunlight. The utilized diodes for these three PV cells in Fig. 5a are D_a_, D_s_, plus D_f_ which are blocking. Here, the captured sunlight irradiations in Fig. 5a for three PV modules are 1000 W/m^2^. From Fig. 5b, in the 1st shaded conditions, the assumed incident sunlight irradiations for the three PV modules are 1000 W/m^2^, 883 W/m^2^, and 783 W/m^2^. Here, the device D_a_ is in ideal state D_s_, plus D_f_ are in the functioning stage. In 2nd shading situation, the captured sunlight irradiations for the modules M_a_, M_b_, plus M_c_ are 1000 W/m^2^, 783 W/m^2^, and 683 W/m^2^. Finally, in the 3rd shading pattern, the capture sunlight irradiations to the PV cells are 1000 W/m^2^, 683 W/m^2^, and 583 W/m^2^. Under three sunlight conditions, the generated PV network characteristics are discussed in Fig. 6. The applied sunlight system parameters are mentioned in Table 1.

**Table 1:** Utilized sunlight system network variables at various sunlight conditions.

S. No	Parameters	Values
1	Evaluated peak power parameter by using optimized method (V_Pm_)	31.04 V
2	Evaluated peak current parameter by using optimized method (I_Pm_)	8.085 amps
3	Evaluated peak power parameter by using optimized method (P_Pm_)	250.98 Watts
4	An evaluated open-circuited voltage of sunlight system (V_OCPV_)	37.12 V
5	Evaluated Short circuited current of Sunlight System (I_SPC_)	8.71 amps
6	Overall sunlight network thermal constant at the uniform voltage	− 0.0321178%/deg.c
7	Overall sunlight network thermal constant at uniform current	0.0447345%/deg.c
8	Functioning ideality constants of Diodes (δ_1_, δ_2_, δ_3_)	0.79, 0.816, & 0.947
9	Obtained sunlight series interfaced resistance of solar module (R_k_)	0.35501 Ώ
10	Obtained sunlight shunt interfaced resistance of solar module (R_q_)	266.012 Ώ
11	PV cells available basic current at diverse irradiations (I_ps_)	8.83310 A
12	Diode generated currents under saturation I_SPCt_, I_SPCy_, I_SPCu_	1.31*10^−10^ A
13	Overall sunlight network collected PV cells (N_s_)	60
14	Selected shading patterns from the sunlight system	4

## Design and development of hybrid MPPT controllers

The sunlight network power capturing depends on the power point tracking methods. Also, it provides more power from the solar strings by moving the operating point of the sunlight network from the local position to the global required region^16^. Here, the major achievement of the sunlight system is a uniform power supply by utilizing the nature-inspired MPPT controller. The MPPT tries to support the sunlight system by supplying a high value of voltages thereby it tries to reach the peak load demand. Here, the modified differential step grey wolf optimization with adaptive fuzzy logic controller is developed for the nonuniform sunlight insolation conditions and it is parallelly investigated along with the advanced recent existing hybrid controllers which are Adaptive Modified Step-P&O (AMS-P&O), Continuous Adjusted Step-Successive Approximation Technique (CAS-SAT), Differential Step Value- Adaptive Neuro-Fuzzy Inference System (DSV-ANFIS), Improved Step with Adaptive Cuckoo Search Technique (IS-ACST), plus Particle Swarm Algorithm based P&O (PSA-P&O). The comprehensive investigation has been carried out by using the following parameters: peak voltage, efficiency of the utilized MPPT, fluctuations of power available at converter load, development cost, plus the functioning speed of the MPP.

### AMS-perturb & observe power point tracking controller

**Figure 7:**
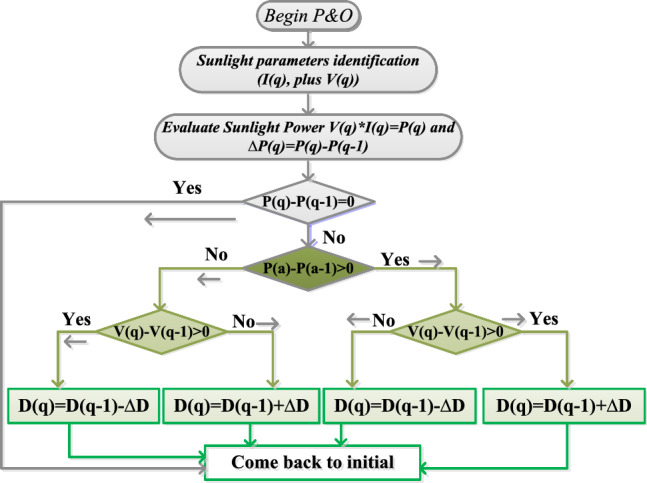
Analysis of AMS-perturb & observe power point tracking controller.

The conventional sunlight-based smart grid power generation network suffers from continuous variation of sunlight temperatures because the overall system stability depends on the interlinked dc-voltage, plus load power. Also, the all-integrated renewable power supply networks supply nonlinear energy that may not be consumed by an industry^17^. So, the P and O concept is interfaced with the sunlight-based microgrid network for running the overall power network at diverse sunlight temperature values. This concept starts functioning by taking the current reference parameter. The adjustment of the sunlight system current has been made in forward movement to run the overall system at peak solar power point. The adjusted current value has a positive indication then the current adjustment has been carried out similarly. Otherwise, the current parameter varies concerning the sunlight power in a reversal direction to function the entire power network at peak load conditions. Here, the sunlight power fed power DC–DC converter works based on the duty signal, which is mentioned in Eq. (8), and Eq. (9). The duty pulses of the converter switch are produced by interfacing the P&O controller to the proposed system. The switching pulses of the converter controlling with the P&O method are illustrated in Fig. 7.

8 $${\text{D}}\left({\text{q}}\right)={\text{D}}\left({\text{q}}-1\right)+\mathrm{adjusted Step}*\left(\frac{{\text{P}}({\text{q}})-{\text{P}}({\text{q}}-1)}{{\text{V}}({\text{q}})-{\text{V}}({\text{q}}-1)}\right)$$

9 $${\text{D}}\left({\text{q}}\right)={\text{D}}\left({\text{q}}-1\right)-\mathrm{adjusted Step}*\left(\frac{{\text{P}}({\text{q}})-{\text{P}}({\text{q}}-1)}{{\text{V}}({\text{q}})-{\text{V}}({\text{q}}-1)}\right)$$

where the variables D(q-1), P(q-1), and V(q-1) are named as storage duty value, sunlight power, plus solar voltage. In an instant, the evaluated sunlight parameters are D(q), P(q), and V(q).

### CAS-SAT power point tracking controller

**Figure 8:**
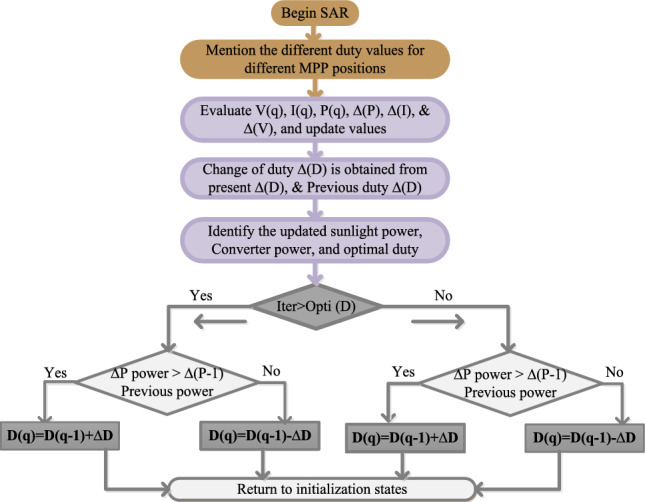
Working of CAS-SAT power point tracking controller.

This type of power point-identifying controller is more useful for traffic-controlling networks. Here, the entire sunlight data is captured at different time intervals then the data is converted into digital form for identifying the proper MPP position^18^. Here, the controller’s Most Significant Bit (MSB) value is given by “1” and then the sequential comparison is applied to each MSB bit. The comparison of MSB and Least Significant Bit (LSB) generates one then the controller MSB is constant until the working point of the sunlight network reaches the global MPP region. Suppose, the comprehensive investigation generates zero value then MSB is initiated “0” and the employed point of sunlight-dependent PV lies on the right-side corner of P–V curve region. This method is more flexible for static sunlight temperature values. Also, its functioning efficiency in bad weather conditions of the sunlight light system is moderate. The continuous improvement step value of the successive approximation controller is discussed in Fig. 8. From Fig. 8, the single switch-based converter supplies the sunlight power with fewer ripples at quick variations of environmental conditions.

### DSV-ANFIS power point tracking controller

**Figure 9:**
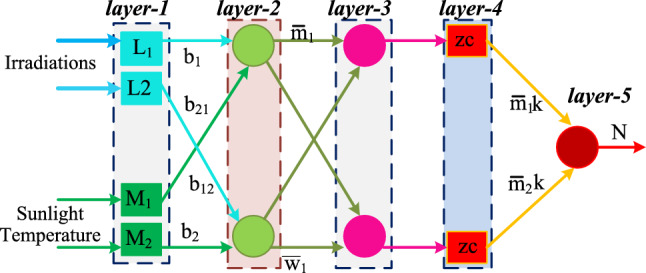
Operational behavior of ANFIS methodology for solar system.

The neural networks are widely utilized for all nonlinear sunlight power production networks because their features are highly efficient in the sunlight system shading patterns identification, easier to design, plus low sensing devices are operated. So, the neural controllers are popularly utilized in facial identification, and financial marketing prediction, highly useful for surveillance applications, plus image processing. Also, the ANN is applied for studying social media users, and aerospace systems. Finally, the neural concept is used to identify the root mean square error of social media networks. From the recent study, the neural controllers are facing the issue of more computational complexity when the selected parameters for this controller are very high. In addition, it needs more time to train all the input constraints. So, the researchers referred to the fuzzy concept for optimizing the training time of the sunlight network. However, the error identification accuracy of this network is low because of the improper utilization of membership values. Moreover, the fuzzy systems do not give the global power of the solar/wind hybrid power network. In^19^, the present researchers apply the ANFIS methodology for the all-renewable energy sources to maintain the uniform grid voltage, and it is implemented from the working principle of fuzzy, plus ANN controllers as shown in Fig. 9. From Fig. 9, the Takagi–Sugeno inference concept is used and its rules are completely depending on the if–then concept. This controller transfers the nonlinear function of the sunlight system into a linear manner for achieving more efficient sunlight power. The rules of this method are finalized by applying the swarm optimization methodology. The sunlight voltage and solar panel voltages are sent to the ANFIS 1st layer which is named “V”, plus “N”. The available signal from the 1st layer is named “S”. The utilized membership parameters for the sunlight system are “K_1_”, “K_2_”, “A_1_”, plus “A_2_” respectively.

10 $$\mathrm{if V is }{{\text{K}}}_{1} ,\mathrm{ plus N is }{{\text{A}}}_{1} ,\mathrm{ then V}={{\text{L}}}_{1}*{\text{z}}+{{\text{M}}}_{1}*{\text{c}}+{{\text{G}}}_{1}$$

11 $$\mathrm{if V is }{{\text{K}}}_{2},\mathrm{ plus N is }{{\text{A}}}_{2},\mathrm{ then V}={{\text{L}}}_{2}*{\text{z}}+{{\text{M}}}_{2}*{\text{c}}+{{\text{G}}}_{2}$$

12 $${{\text{S}}}_{1,{\text{f}}}={\upmu }_{{\text{k}}1}\left({\text{z}}\right)+{\upmu }_{{\text{k}}2}\left({\text{c}}\right);\mathrm{ f}=\mathrm{1,2}$$

13 $${{\text{S}}}_{1,{\text{f}}}={\upmu }_{{\text{a}}1}\left({\text{z}}\right)+{\upmu }_{{\text{a}}2}\left({\text{c}}\right);\mathrm{ f}=\mathrm{1,2}$$

14 $${\uppsi }_{{\text{f}}}\left({\text{z}}\right)=\frac{1}{1+{\left|\frac{{\text{z}}-{{\text{m}}}_{{\text{f}}}}{{{\text{n}}}_{{\text{k}}}}\right|}^{2{{\text{b}}}_{{\text{q}}}}}$$

where the terminologies L_1_, M_1_, G_1_, L_2_, plus M_2_ are identified as the ANFIS supply and subsequent variables. The parameters b_1_, b_2_, $\overline{{{\text{w}} }_{1}}$, plus $\overline{{{\text{m}} }_{1}}$ are the 2nd and 3rd layers weights. The membership constants of ANFIS are ψ, plus k.

### IS-ACST controller for partial shaded sunlight system

**Figure 10:**
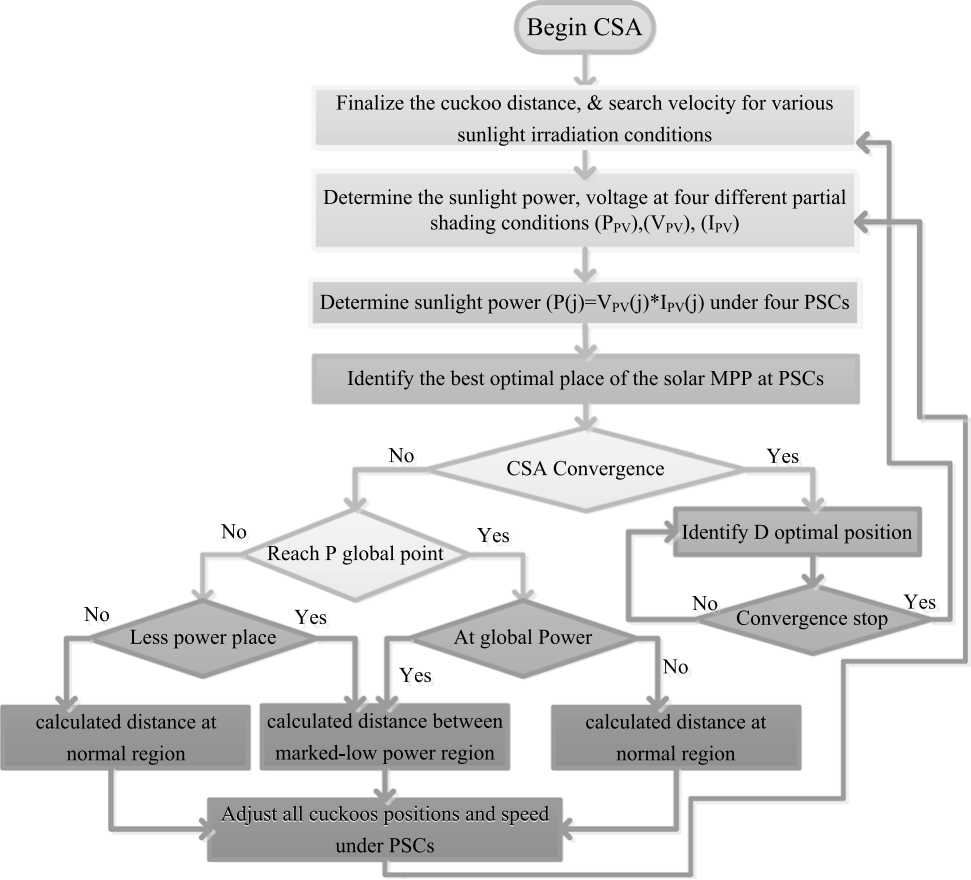
Improved step with adaptive cuckoo search technique for partially shaded PV system.

The cuckoo search concept is selected in^20^ for the optimal solution of a nonlinear photovoltaic sunlight system. The ACS technology is implemented from the various groups of birds. The Levy terminology is the major factor in the adaptive CST because the step length on the sunlight P–V curve is represented by utilizing the Levy flight. Here, the cuckoo agents’ associated weights are prepared with the help of a random search concept, plus probability methodology. The overall search region of the V–I curve is separated into three major parts which are named as medium power place, less power region, plus more power place. From Fig. 10, all the cuckoos start identifying the optimal MPP place with various velocities, plus various distances. In the 1st iteration, the cuckoo’s running direction is completely different. After certain iterations, the cuckoos start running towards the targeted position. The sunlight system functioning accuracy is good in this controller when associated with the ANFIS, plus P&O methods because different cuckoo agents consist of various step values. Also, the utilized cuckoos in this algorithm should be good quality then only it gives high-quality eggs. After the 1st iteration, all cuckoos must and should supply optimal MPP locations. Otherwise, the functioning of the sunlight-dependent cuckoo search controller stops working for the cloudy conditions. The functioning of this algorithm and its global MPP identification is mentioned in Fig. 10. The cuckoo step adjustment and its associated duty value are defined in Eq. (15), plus (16). The levy value of this algorithm is mentioned in Eq. (17).

15 $${\mathrm{step value }}_{{\text{j}}}={\mathrm{step value}}_{{\text{min}}}+\left({\mathrm{step value }}_{{\text{max}}}-{\mathrm{step value}}_{{\text{min}}}\right){{\text{D}}}_{{\text{j}}}$$

16 $${{\text{D}}}_{{\text{j}}}=\left\{\begin{array}{cc}\frac{({{\text{V}}}_{{\text{j}}}-{{\text{V}}}_{{\text{jn}}})}{{{\text{D}}}_{{\text{maximum}}}}& \mathrm{Less power region}\\ 1.50& \mathrm{Moderate Power region}\\ 0& \mathrm{More power region}\end{array}\right.$$

17 $${\text{Levy value}}\left( {\beta {\text{eta}}} \right) = {\text{Levy}}^{{ - \beta {\text{eta}}}} \;0.5 < \beta {\text{eta}} < 3.5$$

where the terms V_j_, V_jn_, plus D_j_ are named as cuckoo local position, global place of the functioning point of the sunlight system, plus duty of the power step-up converter. Finally, the variable $\upbeta$ is helpful for the cuckoo’s step value selection and it is finalized in between 0.5 to 3.5.

### PSA-P&O power point identifier for various PSCs of the sunlight network

The perturb and observe methodology alone generates more distortions in the entire fuel stack/wind power generation network. Also, the functioning of a converter for dual renewable systems is a highly complex task and P&O does not support the flexible operation of this hybrid network. So, many authors studied the combination of P&O with nature-inspired MPPT controllers. In the article^21^, the P&O block is interlinked with the adjustable step involved PSO module for running the hybrid wind/solar power distribution network. Here, at 1st functioning state, the P&O captures the wind velocity, plus sunlight insolation to find out the LMPP positions to optimize its tracking speed and reduce the convergence rate. Once the LMPP position searching is over then the PSA collects the information from the P&O block regarding LMPP places. The PSA functioning starts with identifying the GMPP place. Here, the GMPP fluctuations, and converter power distortions are suppressed by involving the PSA methodology. Due to these features, this hybrid PSA-P&O is applied to stationary sunlight power networks for flexible power utilization. In this standalone network, there are five power devices interlinked with the interleaved power converter, bidirectional, plus quadratic converters for maintaining the uniform voltage in the microgrid network. All three converters’ switching pulses are obtained by focusing on the battery state of charge, plus the state of discharge conditions. The particle agents searching position, and their associated velocities are mentioned in Eq. (18), and Eq. (19).

18 $${{\text{S}}}^{{\text{f}}+1}={\text{W}}*{{\text{S}}}_{{\text{p}}}^{{\text{f}}}+{\uppsi }_{{\text{Q}}}*{\upmu }_{{\text{Q}}}\left({{\text{P}}}_{{\text{best}}}-{{\text{J}}}_{{\text{P}}}^{{\text{f}}}\right)+{\uppsi }_{{\text{R}}}*{\upmu }_{{\text{R}}}({{\text{G}}}_{{\text{best}}}-{{\text{J}}}_{{\text{p}}}^{{\text{f}}})$$

19 $${\text{Y}}^{{{\text{f}} + 1}} = {\text{Y}}_{{\text{p}}}^{{\text{f}}} + {\text{S}}_{{\text{p}}}^{{\text{f}}}$$

where the first iteration, plus complete iterations-based sunlight powers are P_best_, plus G_best_. Similarly, the parameters S, p, f, W, Y, ${\uppsi }_{{\text{Q}}}$, plus ${\upmu }_{{\text{Q}}}$ are named as running velocity of particles, algorithm iteration value, particle number, each particle weight, position of the particle, plus empirical constants.

### Proposed MDSGWA-AFLC power point finding controller

From the complete study above algorithms, the sunlight systems work very effectively at uniform sunlight temperature conditions. But in practical circumstances, the sunlight intensity is not uniform and it disturbs the power system with high fluctuated voltages. At this condition, the sunlight network performance is optimized by proposing the modified GWA-influenced fuzzy methodology for working the entire network at the global peak power point region. Here, the grey wolf MPPT block is designed from the regular phenomena of wolves which are mimics of hunting, and its social leadership. The variables α, β, δ, plus ω are named as leader wolf best solution, 2nd, 3rd, and 4th wolf optimal solution for the nonlinear issue. Here, the wolf takes more time duration for the identification GMPP place. Also, it takes high computational time because multiple iterations are needed to obtain the peak power from the sunlight PV network. So, the wolf distance is adjusted with the help of the linear tuning function which is mentioned as ($\overline{{\text{L}} })$. As a result of this tuning function, the grey wolf controller’s proper use, plus its searching mechanism are balanced. In this improved grey wolf methodology, the search space, plus the total number of agents are balanced for increasing the tracking speed of MPP. The searching velocity of the grey wolf is varied by considering Eq. (20), and its associated distance is changed by the use of Eq. (21). From Eq. (20), and Eq. (21), the term $\overline{{R }_{Position}}(q+1)$ is named as the updated place of the particular wolf “q”. From Eq. (20), the wolf constraints are $\Psi_{1} \in \left( {0,1} \right), \Psi_{2} \in \left( {0,1} \right)$. Similarly, the terminologies L, plus n are the moving length of the wolf and its iteration.

20 $$\overline{{{\text{R}}_{{{\text{Position}}}} }} \left( {{\text{q}} + 1} \right) = \Psi_{1} {*}\overline{{{\text{R}}_{{{\text{Position}}}} }} \left( {{\text{q}} + 1} \right) + \Psi_{2} {*}\left( {{\overline{\text{q}}}^{1} - {\overline{\text{q}}}} \right)$$

21 $$\overline{{\text{L}} }\left({\text{n}}\right)={{\text{L}}}_{{\text{intial}}}-\left({{\text{L}}}_{{\text{intial}}}-{{\text{L}}}_{{\text{final}}}\right)\left(\frac{{\text{Mait}}-{\text{n}}}{{\text{Mait}}}\right)$$

**Figure 11:**
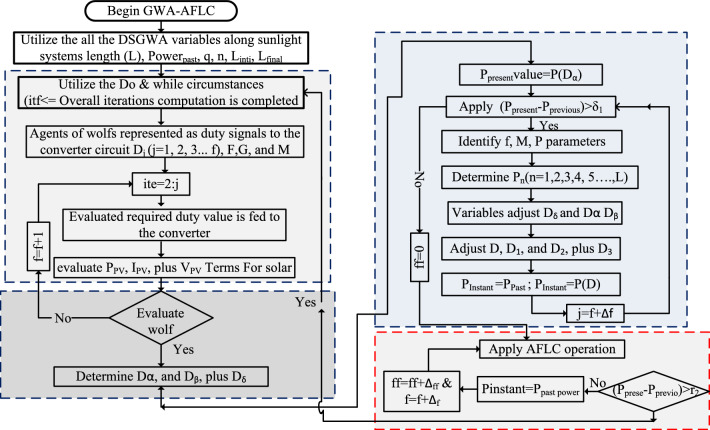
Proposed grey wolf technology optimized fuzzy controller for MPP tracking.

The above strategy works until the 2 local MPPs search region. After that, the fuzzy methodology is utilized to reach the peak power position. Here, the fuzzy membership selection is the major issue because the improper membership selection creates more convergence time, plus excessive oscillations of the PV power. So, the GWA working strategy is applied to the fuzzy system for suitable membership function selection. After that, the global present power is equated with the past global power for identifying the suitable error factor to the fuzzy system as given in Eq. (22), plus Eq. (23).

22 $$\left|{{\text{Power}}}_{{\text{Instant}}}-{{\text{Power}}}_{{\text{past}}}\right|\le {\mathrm{\S }}_{1}$$

23 $$\left|{{\text{Power}}}_{{\text{Instant}}}-{{\text{Power}}}_{{\text{past}}}\right|\ge {\mathrm{\S }}_{1}$$

where the terms Power_Instant_, and Power_past_ are the sunlight network power at time t = 0, and Δt = t − 1. The term ${\S }_{1}$ utilized for error signal identification of fuzzy and it is equal to 0.059. From this 0.059 value, the fuzzy generated signal is sent to the grey wolf block as shown in Fig. 11. Finally, the term ${\S }_{2}$ is sent to the grey wolf network for collecting the miscalculation constant.

## Development of DC–DC power transfer converter

**Figure 12:**
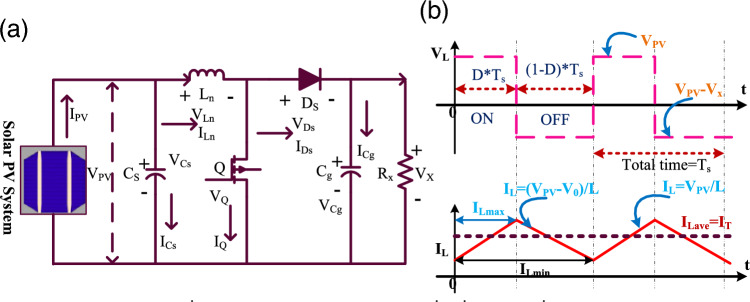
Selected sunlight power based, (**a**) Converter circuit, and (**b**) its power waveforms.

The sunlight network power supply efficiency completely depends on the DC–DC power converter. In the review^22^, the authors explained the various high voltage conversion ratio-based z-source, dual-phase interleaved, plus quadratic converters for stabilizing the DC-link voltage in the hybrid electric vehicle network. The z-source circuit in the conventional DC–DC circuit creates more heating, plus conduction loss. Also, it may not be installed in the rural power generation networks. The z-source circuit suffers from more inductive ripples and more development costs. The triple-phase quadratic converter is integrated with the battery-dependent fuel stack network for uniform power distribution to the industrial networks. In this converter circuit, the utilized inductors are six, switches five, plus eight capacitors. As a result, the entire renewable power system design requirements, plus complexity are increased. In the article^23,24^, the researchers integrated the three renewable networks which are solar, battery, plus wind power networks for the uniform power distribution to the electric vehicle charging stations. In this power network, the dual-direction power electronics converters are interlinked with the common DC-bus for supplying the energy to the urban area people, and industries. However, these converter circuits needed more passive, and semiconductor elements. As a result, these types of converter circuits understanding and development is more difficult. So, the single switch power transfer circuit is selected in this work for giving the energy to the electric battery charging network. This circuit takes very few components for implementation. The power transmission losses in this converter system are moderate. Here, from Fig. 12, the sunlight energy is sent to the selected power converter to improve the power supply ability to the resistive element. From Fig. 12b, the inductor current starts moving from origin to peak value at the time of switch functioning state. The converter circuit gives enhanced sunlight system voltage which is explained in Eq. (27).

24 $${{\text{Duty}}*{\text{T}}}_{{\text{s}}}{{\text{V}}}_{{\text{x}}}+(\left({{\text{V}}}_{{\text{x}}}-{{\text{V}}}_{{\text{PV}}}\right)\left(1-{\text{Duty}}\right){{\text{T}}}_{{\text{s}}}=0$$

25 $$-{{\text{I}}}_{{\text{PV}}}{\text{Duty}}*{{\text{T}}}_{{\text{s}}}+(\left({{\text{I}}}_{{\text{x}}}-{{\text{I}}}_{{\text{PV}}}\right)\left(1-{\text{Duty}}\right){{\text{T}}}_{{\text{s}}}=0$$

26 $${\text{V}}_{{{\text{PV}}}} = {\text{V}}_{{\text{x}}} /\left( {1 - {\text{Duty}}} \right),\& {\text{ I}}_{{{\text{PV}}}} = {\text{I}}_{{\text{x}}} \left( {1 - {\text{Duty}}} \right)$$

27 $$\frac{{{\text{V}}}_{{\text{PV}}}}{{{\text{I}}}_{{\text{PV}}}}=\frac{{{\text{V}}}_{{\text{x}}}}{{{\text{I}}}_{{\text{x}}}}\left(\frac{1}{{(1-{\text{Duty}})}^{2}}\right)$$

28 $$\frac{{{\text{V}}}_{{\text{PV}}}}{{{\text{I}}}_{{\text{PV}}}}={{\text{R}}}_{{\text{PV}}} \& \frac{{{\text{V}}}_{{\text{x}}}}{{{\text{I}}}_{{\text{x}}}}={{\text{R}}}_{{\text{x}}}$$

29 $${{\text{R}}}_{{\text{x}}}={{\text{R}}}_{{\text{PV}}}\left({(1-{\text{Duty}})}^{2}\right)$$

## Detailed discussion of simulation results

The proposed modified grey wolf technology-based adaptive FLC is developed by selecting the MATLAB environment. Here, the AFLC membership functions values selection and its working behavior depending on the MDSGWA controller, plus solar network irradiations occurrence angle. The utilized parameter C_s_ value is 238 µF which suppresses any distortions in the sunlight network voltages. Also, it tries to maintain the uniform source power irrespective of sunlight temperature conditions. In addition, the element Cs balance the quick variation of solar system output voltage. The selected value of the PV side inductor L_n_ is 2.24 mH, and it smoothest the PV stack current at quick deviations of the sunlight system insolation, plus temperatures. The inductor stores the source energy by operating the Metal Oxide Semiconductor Field Effect Transistor (MOSFET) switch. The merits of this switch are simple parallel operation, medium sensitivity with the sunlight temperature, low-level transconductance effect, functioning temperature ability of more than 200 °C, moderate switching pulse driver design complexity, ability to function at more operating frequencies, plus less power consumption. Finally, the utilized element C_g_ value is 240 µF which can maintain the consumer voltage uniform under diverse atmospheric conditions. The resistor R_x_ is placed near the load capacitor for capturing the sunlight power.

### Results analysis of proposed sunlight system at 1st shading (1000 W/m^2^, 883 W/m^2^, and 783 W/m^2^)

**Figure 13:**
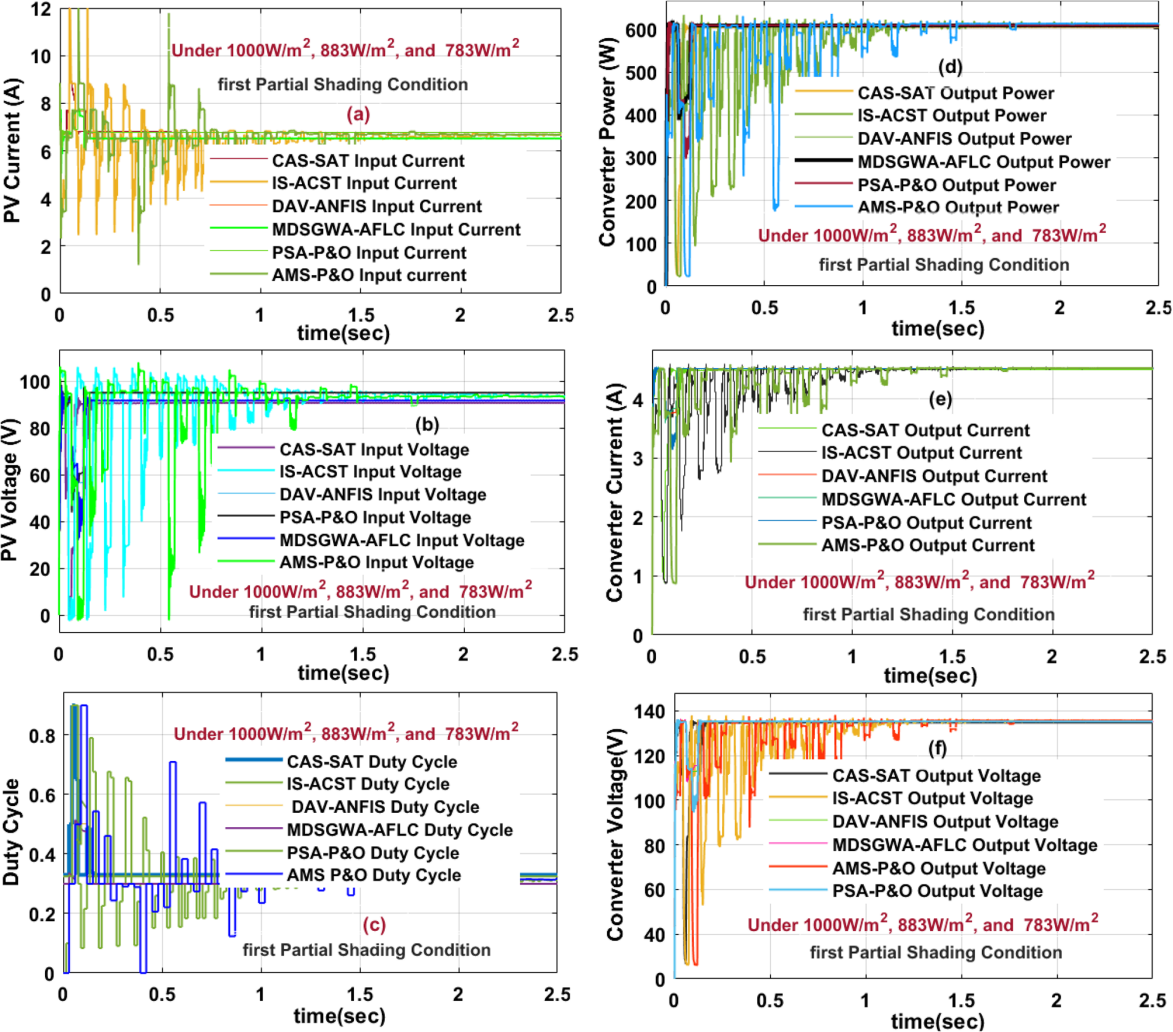
Sunlight system and single switch power converter current, voltage, duty cycle, power at 1000 W/m^2^, 883 W/m^2^, and 783 W/m^2^.

The sunlight network generated nonlinear voltage is mentioned in Fig. 6. From Fig. 6, for the uniform sunlight values, the captured PV power, plus solar voltage is 752.94 W, and 93.12 V. Suddenly, the sunlight network captured irradiations are reduced to 1000, 883, and 783 W/m^2^ at 25 °C then the availed power, solar current, plus sunlight system voltages are 631.55 W, 7.01 A, and 90.2 V respectively. Here, the captured power is reduced due to the shading phenomena and there is the chance that the operating point of the sunlight system may settle at any local peak voltage points which are defined as LMPP^1^, plus LMPP^2^. In this shading pattern, the availed sunlight network current, solar voltage, plus converter functioning duty cycle are given in Fig. 13a, b, plus 13c. The MDSGWA-AFLC, plus PSA-P&O fed sunlight system, and power converter network produced voltages, powers, efficiency’s, currents, plus duty cycles are 88.19 V, 140.22 V, 615.66 W, 607.42 W, 98.66%, 6.981 A, 4.3319 A, 0.72, 88.379 V, 140.1 V, 616.36 W, 606.81 W, 98.45%, 6.974 A, 4.3312 A, plus 0.71 respectively.

At 1000 W/m^2^, 883 W/m^2^, and 783 W/m^2^, the DC–DC circuit supplying settling power time by selecting the AMS-P&O, CAS-SAT, DAV-ANFIS, IS-ACST, PSA-P&O, plus MDSGWA-AFLC MPPT controllers are 0.0318 s, 0.0283 s, 0.027 s, 0.023 s, 0.021 s, 0.0197 s respectively, and its associated duty values are 0.72, 0.734, 0.74, 0.751, 0.75, plus 0.78. The extracted converter, plus sunlight network powers, voltages, plus current values by interfacing the at this first shading condition AMS-P&O, CAS-SAT, DAV-ANFIS, plus IS-ACST are 578.91 W, 597.98 W, 137.422 V, 87.79 V, 4.212 A, 6.811 A, 590.17 W, 602.27 W, 138.307 V, 87.641 V, 4.2671 A, 6.872 A, 594.89 W, 607.03 W, 138.452 V, 88.001 V, 4.2967 A, 6.898 A, 598.55 W, 608.83 W, 139.01 V, 88.07 V, 4.3058 A, plus 6.913 A respectively. The proposed MDSGWA-AFLC involves moderate design complexity and supplies fewer fluctuations in the overall sunlight network. This proposed MPPT block requires very less iterations for finding the accurate membership functions of the fuzzy block. However, the output power supply ability of the sunlight network under partial shading is low when associated with uniform sunlight insolation conditions. The converter load power, current, and its supplied voltage to the consumers are discussed in Fig. 13d, e, plus 13f.

### Results analysis of proposed sunlight system at 2nd shading (1000 W/m^2^, 783 W/m^2^, and 683 W/m^2^)

**Figure 14:**
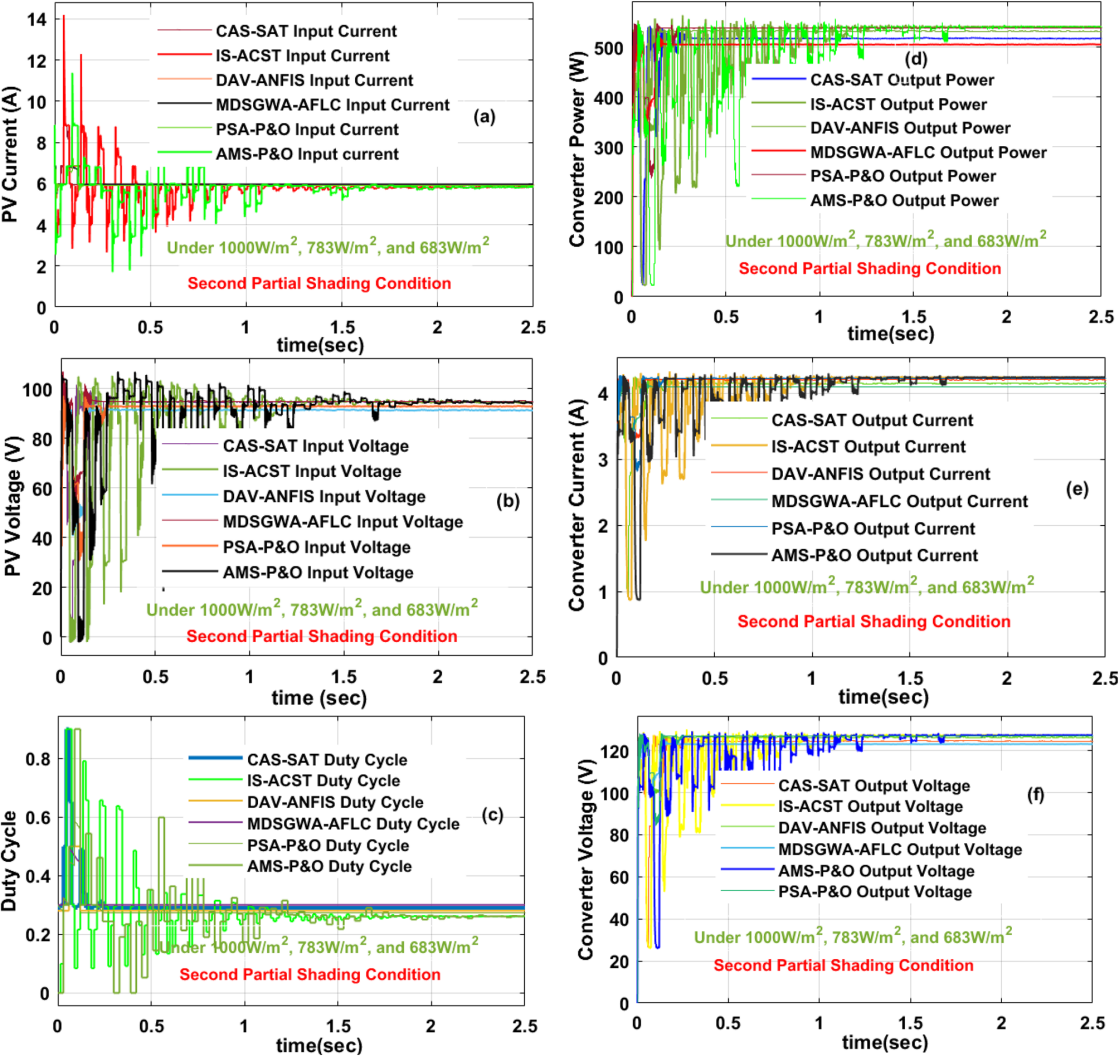
Sunlight system and single switch power converter current, voltage, duty cycle, power at 1000 W/m^2^, 783 W/m^2^, and 683 W/m^2^.

Similar to the first shading effect on the sunlight system, at the second shading condition (1000 W/m^2^, 783 W/m^2^, and 683 W/m^2^), the AMS-P&O, CAS-SAT, DAV-ANFIS, IS-ACST, PSA-P&O, plus MDSGWA-AFLC fed converter, and sunlight system supplied current, load voltage, power, and its duty values are 4.0207 A, 6.129 A, 127 V, 86.227 V, 510.63 W, 528.49 W, 0.79, 4.1632 A, 6.157 A, 127.03 V, 88.496 V, 528.86 W, 544.87 W, 0.8, 4.143 A, 6.198 A, 127.99 V, 87.855 V, 530.30 W, 544.53 W, 0.81, 4.15813 A, 6.227 A, 128.18 V, 87.389 V, 532.99 W, 544.22 W, 0.74, 4.146 A, 6.282 A, 129.79 V, 87.406 V, 538.11 W, 549.09 W, 0.761, 4.1547 A, 6.313 A, 129.88 V, 87.121 V, 539.62 W, 550.01 W, plus 0.78 respectively. The available solar current, supplied voltage, converter duty signal, load power, load current plus load voltages are expressed in Fig. 14a, b, c, d, e, plus 14f. Here, in this second shading pattern, the captured solar irradiation values are still reduced. As a result, the consumer collected voltage value is reduced when associated with the uniform, plus the first shading condition of the solar PV network. The development complexity of sunlight-based DAV-ANFIS, IS-ACST, plus PSA-P&O controllers is quite high when correlated with the AMS-P&O, CAS-SAT and it is compensated in the form of operating efficiency, and settling time of the load power which are equal to 97.77%, 0.18 s, 97.88%, 0.098 s, 98%, 0.034 s, 96.62%, 0.25 s, 97.06%, plus 0.21 s respectively. Here, the grey wolf methodology takes more iterations for identifying the fuzzy membership values. The tracking speed of the sunlight system functioning operating point is very high by applying the IS-ACST controller by relating with the AMS-P&O, plus CAS-SAT power point identifying controllers. From Fig. 14f, the proposed MPPT block captures more sunlight intensity, and it tries to maximize the overall system functioning efficiency.

### Results analysis of proposed sunlight system at 3rd shading (1000 W/m^2^, 683 W/m^2^, and 583 W/m^2^)

**Figure 15:**
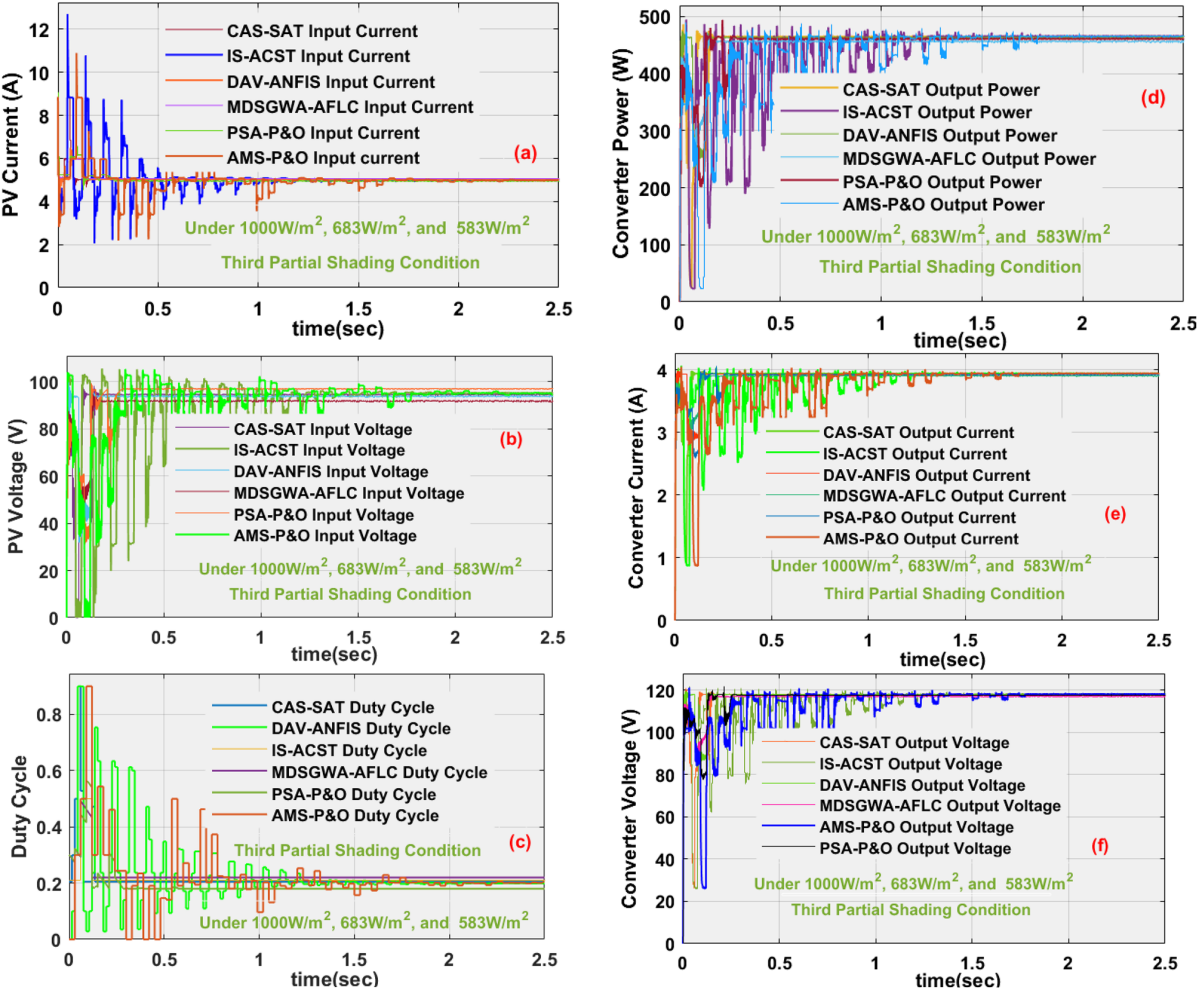
Sunlight system and single switch power converter current, voltage, duty cycle, power at 1000 W/m^2^, 683 W/m^2^, and 583 W/m^2^.

In this 3rd shading pattern condition, the assumed sunlight intensity is again reduced, and its related solar system-rated power is reduced when relating to the 1st, plus 2nd shading conditions. In 3rd shading pattern, the captured sunlight irradiations are 1000 W/m^2^, 683 W/m^2^, and 583 W/m^2^. Here, the sunlight current, voltage of the PV, PV power, converter current, load voltage, plus resistive power by employing the AMS-P&O, CAS-SAT, DAV-ANFIS, IS-ACST, PSA-P&O, plus MDSGWA-AFLC are 5.413 A, 86.67 V, 469.17 W, 3.8083 A, 118.27 V, 450.41 W, 5.411 A, 87.182 V, 474.36 W, 3.84046 A, 118.78 V, 456.17 W, 5.482 A, 86.14 V, 472.27 W, 3.83471 A, 119.25 V, 457.29 W, 5.491 A, 86.028 V, 472.38 W, 3.8223 A, 119.85 V, 458.11 W, 5.51 A, 86.022 V, 474.02 W, 3.8499 A, 119.48 V, 459.99 W, 5.527 A, 85.941 W, 475 W, 3.8407 A, 120.2 V, plus 461.66 W. The sunlight-produced currents and their voltages are extracted by interfacing the proposed modified grey wolf concept along with the resistive load as given in Fig. 15a, plus 15b. From Fig. 15c, d, e, and Fig. 15f, the obtained duty signal values, and the DC–DC circuit supplying settling power time by using the AMS-P&O, CAS-SAT, DAV-ANFIS, IS-ACST, PSA-P&O, plus MDSGWA-AFLC are 0.37, 0.3 s, 0.42, 0.28 s, 0.4, 0.22 s, 0.39, 0.09 s, 0.421, plus 0.031 s respectively, and its associated power production network efficiencies are 96%, 96.16%, 96.21%, 97.01%, 97.04%, plus 97.19%. The overall system analysis is mentioned in Table 2.

**Table 2:** Overall sunlight system performance parameters by interfacing the hybrid MDSGWA-AFLC MPPT controllers.

Method	Current of solar	Voltage of solar	Power of solar	A current of power converter	Voltage of power converter	Load power	Efficiency of controller	Converter power settling time	Fluctuations of solar power	Complexity in controller
Sunlight system at first shading condition (1000 W/m^2^, 883 W/m^2^, plus 783 W/m^2^)										
AMS-P&O	6.811 A	87.79 V	597.98 W	4.21264 A	137.422 V	578.91 W	96.821%	0.0318 s	More	Low
CAS-SAT	6.872 A	87.641 V	602.27 W	4.26710 A	138.307 V	590.17 W	97.99%	0.0283 s	More	Low
DAV-ANFIS	6.898 A	88.001 V	607.03 W	4.2967 A	138.452 V	594.89 W	98.021%	0.027 s	Medium	Low
IS-ACST	6.913 A	88.070 V	608.83 W	4.3058 A	139.01 V	598.55 W	98.31%	0.023 s	Less	Medium
PSA-P&O	6.974 A	88.379 V	616.36 W	4.3312 A	140.10 V	606.81 W	98.45%	0.021 s	Less	Medium
MDSGWA-AFLC	6.981 A	88.19 V	615.66 W	4.3319 A	140.22 V	607.42 W	98.66%	0.0197 s	Less	Medium
Sunlight system at first shading condition (1000 W/m^2^, 783 W/m^2^, plus 683 W/m^2^)										
AMS-P&O	6.129 A	86.227 V	528.49 W	4.0207 A	127.00 V	510.63 W	96.62%	0.25 s	More	Low
CAS-SAT	6.157 A	88.496 V	544.87 W	4.1632 A	127.03 V	528.86 W	97.06%	0.21 s	More	Low
DAV-ANFIS	6.198 A	87.855 V	544.53 W	4.14329 A	127.99 V	530.30 W	97.77%	0.18 s	Medium	Low
IS-ACST	6.227 A	87.389 V	544.22 W	4.15813 A	128.18 V	532.99 W	97.88%	0.098 s	Less	Medium
PSA-P&O	6.282 A	87.406 V	549.09 W	4.1460 A	129.79 V	538.11 W	98.00%	0.034 s	Less	Medium
MDSGWA-AFLC	6.313 A	87.121 V	550.01 W	4.1547 A	129.88 V	539.62 W	98.11%	0.027 s	Less	Medium
Sunlight system at first shading condition (1000 W/m^2^, 683 W/m^2^, plus 583 W/m^2^)										
AMS-P&O	5.413 A	86.67 V	469.17 W	3.80831 A	118.27 V	450.41 W	96.00%	0.3 s	More	Low
CAS-SAT	5.441 A	87.182 V	474.36 W	3.84046 A	118.78 V	456.17 W	96.16%	0.28 s	More	Low
DAV-ANFIS	5.482 A	86.14 V	472.27 W	3.83471 A	119.25 V	457.29 W	96.21%	0.22 s	Medium	Low
IS-ACST	5.491 A	86.028 V	472.38 W	3.8223 A	119.85 V	458.11 W	97.01%	0.09 s	Less	Medium
PSA-P&O	5.510 A	86.022 V	474.02 W	3.8499 A	119.48 V	459.99 W	97.04%	0.039 s	Less	Medium
MDSGWA-AFLC	5.527 A	85.941 V	475.00 W	3.84072 A	120.20 V	461.66 W	97.19%	0.031 s	Less	Medium

## Conclusion

The modified differential step grey wolf controller with adaptive fuzzy logic system is designed and it is analyzed at rapid changes of the sunlight intensity conditions by selecting the MATLAB/Simulink network. From the simulative results investigation, it has been identified that the sunlight system power production is more at constant irradiation situations, and it supplies low-level power when the incident irradiations are gradually decreased. However, the sunlight-dependent PV array power utilization is reduced in cloudy environmental conditions. So, the hybrid MDSGWA-AFLC is developed for extracting more peak power from the solar system at diverse environmental cloudy conditions. This proposed controller’s functioning efficiency is higher for the PV array when equaled with the AMS-P&O, CAS-SAT, DAV-ANFIS, IS-ACST, and PSA-P&O controller. In addition, it takes very little time to identify the MPP position of solar PV. The merits of this proposed controller are less iterations needed for identifying the fuzzy controller membership values, good reliability, less converter power settled time, plus the ability to work at uniform and partially shaded environmental conditions. Here, the PV-generated voltage is improved by integrating the DC–DC single-switch power converter. This converter needed very few passive components for the implementation, had low installation cost, was more reliable, plus needed very little space for mounting.

### Supplementary Information

Supplementary Information.

## Data Availability

The datasets used and/or analyzed during the current study available from the corresponding author on reasonable request.
